# Daily Rhythms of Plasma Melatonin, but Not Plasma Leptin or Leptin mRNA, Vary between Lean, Obese and Type 2 Diabetic Men

**DOI:** 10.1371/journal.pone.0037123

**Published:** 2012-05-18

**Authors:** Simone Mäntele, Daniella T. Otway, Benita Middleton, Silvia Bretschneider, John Wright, M. Denise Robertson, Debra J. Skene, Jonathan D. Johnston

**Affiliations:** Faculty of Health and Medical Sciences, University of Surrey, Guildford, United Kingdom; Morehouse School of Medicine, United States of America

## Abstract

Melatonin and leptin exhibit daily rhythms that may contribute towards changes in metabolic physiology. It remains unclear, however, whether this rhythmicity is altered in obesity or type 2 diabetes (T2DM). We tested the hypothesis that 24-hour profiles of melatonin, leptin and leptin mRNA are altered by metabolic status in laboratory conditions. Men between 45–65 years old were recruited into lean, obese-non-diabetic or obese-T2DM groups. Volunteers followed strict sleep-wake and dietary regimes for 1 week before the laboratory study. They were then maintained in controlled light-dark conditions, semi-recumbent posture and fed hourly iso-energetic drinks during wake periods. Hourly blood samples were collected for hormone analysis. Subcutaneous adipose biopsies were collected 6-hourly for gene expression analysis. Although there was no effect of subject group on the timing of dim light melatonin onset (DLMO), nocturnal plasma melatonin concentration was significantly higher in obese-non-diabetic subjects compared to weight-matched T2DM subjects (*p*<0.01) and lean controls (*p*<0.05). Two T2DM subjects failed to produce any detectable melatonin, although did exhibit plasma cortisol rhythms comparable to others in the group. Consistent with the literature, there was a significant (*p*<0.001) effect of subject group on absolute plasma leptin concentration and, when expressed relative to an individual’s 24-hour mean, plasma leptin showed significant (*p*<0.001) diurnal variation. However, there was no difference in amplitude or timing of leptin rhythms between experimental groups. There was also no significant effect of time on leptin mRNA expression. Despite an overall effect (*p*<0.05) of experimental group, post-hoc analysis revealed no significant pair-wise effects of group on leptin mRNA expression. Altered plasma melatonin rhythms in weight-matched T2DM and non-diabetic individuals supports a possible role of melatonin in T2DM aetiology. However, neither obesity nor T2DM changed 24-hour rhythms of plasma leptin relative to cycle mean, or expression of subcutaneous adipose leptin gene expression, compared with lean subjects.

## Introduction

The circadian timing system regulates many aspects of physiology and circadian disruption is linked to multiple metabolic diseases, including type 2 diabetes mellitus (T2DM) [1], [2]. In mammals, the master circadian clock is located within the suprachiasmatic nuclei (SCN) of the hypothalamus. This SCN clock is synchronised to the external light-dark cycle and acts to maintain the correct phasing of clocks in other brain areas and in most peripheral tissues [3]. In human chronobiology studies, endogenous circadian phase is commonly defined by measuring SCN-driven rhythms in the plasma concentration of hormones, such as melatonin and cortisol.

It is now clear that there is a strong interaction between multiple aspects of circadian and metabolic physiology. For example, tissue-specific disruption of the murine liver or pancreatic circadian clock has adverse effects on whole body glucose regulation [4]–[6]. Circadian clocks have also been identified in white adipose tissue (WAT), another key metabolic tissue that is intimately linked to glucose regulation and the risk of developing T2DM [7], [8]. Robust daily rhythms of WAT gene expression have been observed in mice, rats and humans [9]–[11]. Moreover, circadian rhythms have also been reported in WAT explants and cultured adipocytes [12]. At present, the function(s) of WAT rhythms are poorly understood, although they may include temporal control over the secretion of the adipose signalling molecules, adipokines [13].

Multiple adipokines exhibit 24-hour plasma rhythms in humans. Of these, the best studied is the prototypic adipokine, leptin, whose daily rhythm is thought to be generated by an interaction between circadian rhythms, feeding and time awake [14], [15]. Leptin rhythms have been proposed to contribute to the daily variation in appetite, and some studies have reported differences in these rhythms in individuals who are obese and/or have T2DM. There are conflicting reports, however, describing leptin rhythmicity in the literature [16]–[18]. These differences may well derive from differences in design of the studies, many of which did not include stringent control of circadian phase.

An important aspect of physiology that interacts with both circadian and metabolic processes is sleep. Restriction of sleep duration is associated with glucose dysregulation, increased appetite and increased body weight in epidemiological and laboratory studies [19]. These effects are believed to be mediated in part by alterations in neuroendocrine function, including increased sympathetic nervous activity, reduced plasma leptin and increased plasma ghrelin concentrations. Careful control of both circadian rhythms and sleep is therefore important for meaningful analysis of metabolism.

In this study, we have tested the hypothesis that daily melatonin and leptin rhythms are different in lean, obese non-diabetic and T2DM men, following strict circadian control. Comparing different biological rhythms within and between individuals requires an endogenous marker of circadian timing. Using dim light melatonin onset (DLMO), the plasma leptin data were normalised to the phase of the melatonin rhythm, considered a reliable circadian marker. Finally, we have used a novel serial biopsy approach to determine daily rhythms of leptin mRNA expression in subcutaneous WAT from the same subjects. Our data reveal differences in melatonin rhythm amplitude, but not in rhythms of plasma leptin or leptin mRNA.

## Methods

### Ethics Statement

All aspects of the study were conducted in accordance with the Declaration of Helsinki and received a favourable ethical opinion from the Surrey Research Ethics Committee and the institutional ethics committee (University of Surrey Ethics Committee). Research participants gave written informed consent before taking part in the study.

### Participants

Twenty five participants aged between 40 and 65 years (53.5±1.3; mean ± SEM) were recruited into 3 age-matched groups (8 lean healthy, 10 obese non-diabetic, and 7 obese T2DM). Recruitment details and calculation of homeostatic model assessment of insulin resistance (HOMA-IR) are described elsewhere [11]. Measurement of insulin, glucose and HbA1c was conducted by the Clinical Biochemistry Department at the Royal Surrey County Hospital, Guildford, UK. Two participants of the T2DM group were diet and exercise controlled and 5 participants were treated with combinations of metformin, statins, ramipril and hypertension medication. The T2DM participants had been diagnosed with T2DM for between 2 and 22 years (7.3 2.7; mean ± SEM). In addition to good diabetes control, T2DM participants reported no other major health problems and so were extremely unlikely to be suffering from autonomic neuropathy. Information about clock and clock-related gene expression in subcutaneous WAT biopsies in the participants can be found elsewhere [11]. Relative to our previous work [11], this study excluded one participant of the T2DM group as well as one participant of the obese non-diabetic group because of insufficient plasma samples.

### Pre-study Week and Laboratory Study

Protocol details of the pre-study week are described elsewhere [11]. In brief, actigraphy, food and sleep diaries were used to ensure that the subjects’ behaviour during the week before the laboratory study was as controlled as possible. All experimental procedures were carried out at the Surrey Clinical Research Centre. Volunteers arrived in the afternoon of day 0 for a night of adaptation. Throughout the laboratory study, they were required to maintain a semi-recumbent posture. They were required to remain awake with lights on between 0630 and 2230 h (440–825 lux in direction of gaze) and allowed to sleep with lights off between 2230 and 0630 h (0 lux). During the waking period, participants were fed with hourly nutritional drinks (Fortisip; Nutricia, Schiphol, The Netherlands) and were allowed to drink water ad libitum. Daily energy intake was basal metabolic rate multiplied by 1.1, divided equally over the waking hours Light exposure, posture and food intake were controlled throughout the 25 hour laboratory sampling period.

### Plasma Melatonin Measurement

Melatonin was measured using a direct radioimmunoassay [20]. The inter-assay coefficients of variation were 25% at 9 pg/ml (n = 13), 15% at 21 pg/ml (n = 21), 17% at 94 pg/ml (n = 16) and 12% at 114 pg/ml (n = 15). The average detection limit was 5.8 0.6 pg/ml (mean SEM). The dim light melatonin onset (DLMO) was calculated using the 25% method [21]. In brief, 25% of the difference between the baseline (mean of six values) and the peak (average of the three highest points) was calculated. This 25% threshold was used to determine the timing of the crossing points of the melatonin rhythm for calculation of the time of DLMO.

### Plasma Cortisol Measurement

Cortisol was measured using a previously validated radioimmunoassay [22]. The inter-assay coefficients of variation were 12% at 97 nM (n = 5), 21% at 348 nM (n = 5) and 15% at 606 nM (n = 5). A cosine wave using the equation shown was fitted to the cortisol data sets using SAS.

### Plasma Leptin Measurement

A commercial human leptin radioimmunoassay kit (Millipore, Watford, UK) was used according to the manufacturer’s instructions. The inter-assay coefficients of variation were 10% at 4 ng/ml (n = 6) and 19% at 15 ng/ml (n = 7). For some analyses, leptin concentration for each individual subject was calculated as % of the average value for that subject. These data were then plotted according to external clock time and to endogenous circadian time using the subject’s DLMO. A cosinor wave was fitted to each individual leptin profile, as described for the plasma cortisol analysis.

### RNA Extraction and Quantification

Subcutaneous adipose tissue biopsies taken from the upper buttock region were washed with saline, snap frozen and then stored at −80C. Total RNA was extracted using TRIZOL according to the manufacturer’s instructions, cDNA was synthesised and leptin mRNA quantified using Taqman quantitative RT-PCR. The sequences of the leptin primer probe set were 5′-3′ TCCTCACCAGTATGCCTTCCA; 3′-5′ GTGAAGAAGATCCCGGAGGTT and the TaqMan probe CGTGATCCAAATATCCAACGACCTGGA. The full method is described in detail elsewhere [11].

### Statistics

Data were analysed by either 1-way or 2-way repeated measures ANOVA, with Bonferroni post-hoc test, or correlation following a linear regression, as appropriate (Prism v5.0, GraphPad Software, San Diego, USA).

## Results

Pre-screen participant data are shown in Table 1. The three subject groups were age matched with no significant (*p*>0.05, 1-way ANOVA) difference between the groups. There were significant (*p*<0.05) overall effects of subject group on all other parameters. Post-hoc analysis revealed that waist circumference and BMI were lower in the lean control group than both other groups, with no difference between obese non-diabetic and T2DM subjects. By contrast, fasting glucose and insulin concentrations, HbA1c and homeostatic model assessment of insulin resistance (HOMA-IR) were highest in the T2DM group, with no difference between the lean and obese non-diabetic groups.

**Table 1:** Pre-screen participant data.

Variable	Leanmean SEM	Obesenon-diabetic	Type 2diabetic
Number	8	10	7
Age [years]	53.8 2.1	50.8 2.9	57.1 1.6
Waist circumference [cm]	88.9 2.3	105.9 1.4*	113.5 3.2*
BMI [kg/m^2^]	23.2 0.5	30.1 0.8*	32.0 0.9*
Fasting ?glucose [mmol/l]	4.2 0.2	4.8 0.3	6.7 0.5**^+^** *
Fasting ?insulin [pmol/l]	28.1 5.9	40.6 6.8	102.1 27.8**^+^** *
HbA1c ?[mmol/mol]	35.0 1.7	35.8 1.6	51.1 3.0**^+^** *
HOMA-IR	0.5 0.1	0.8 0.1	2.0 0.6**^+^** *

* *P*<0.05 compared to lean participants; ^+^ *P*<0.05 compared to obese non-diabetic participants (1-way ANOVA with Bonferroni post-hoc test).

Compliance with the prescribed pre-laboratory sleep-wake schedule was checked by actigraphy; in all cases, actigrams showed a sharp onset of activity at 06∶30 h and a sharp drop of activity at 22∶30 h (data not shown). These findings were corroborated by the timing of morning (06∶33 h 2 min) and evening (22∶28 h 7 min) voice mail recordings, together with analyses of the sleep diaries (get up time 06∶45 h 5 min; try to sleep time 22∶44 h 4 min; all times represent mean SEM). Food diaries confirmed that the participants followed the dietary restrictions in the week prior the study.

**Figure 1:**
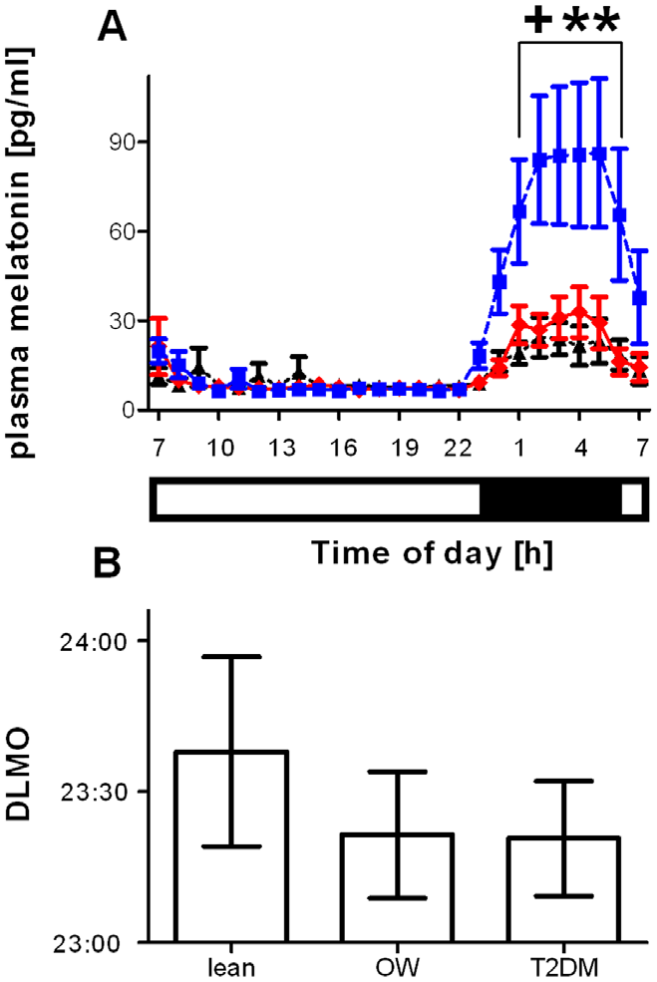
Differences in amplitude, but not onset time, of nocturnal plasma melatonin concentration. (a) Data in the top panel represent mean ± SEM of plasma melatonin concentrations over 25 hours. Diamonds, solid red line  =  lean subjects (n = 8); squares, dashed blue line  =  obese non-diabetic subjects (n = 10); triangles, dotted black lines  =  type 2 diabetic subjects (n = 7). The light-dark conditions are indicated by the bar below the x-axis. There was a significant (*p*0.001; 2-way repeated measures ANOVA) effect of time, group and time x group interaction. Nocturnal melatonin concentrations were significantly higher in the obese non-diabetic group (^+^ *p*<0.05, vs lean, **p<0.01 vs type 2 diabetic subjects). (b) Data in the bottom panel represent mean ± SEM of the dim light melatonin onset (DLMO) in each group. There was no significant (*p*>0.05, 1-way ANOVA) difference between the group averages. Lean  =  lean healthy participant group; ow  =  obese non-diabetic group; T2DM  =  type 2 diabetic group.

Melatonin rhythms were detected in all lean and obese non-diabetic subjects and five of the seven T2DM subjects (Figure S1, S2, and S3). There was a significant (*p*<0.001; 2-way repeated measures ANOVA) effect of time and subject group, together with a significant time x group interaction on plasma melatonin concentrations (Figure 1a). Statistical significance was also maintained if only values during the dark phase were analysed (data not shown). Nocturnal melatonin concentrations in the obese non-diabetic group were significantly higher than in the lean (*p*<0.05) and T2DM (*p*<0.01) groups, despite no difference in BMI or waist circumference between the obese non-diabetic and T2DM subjects. Within the obese non-diabetic and T2DM groups, there was no significant association between BMI and melatonin concentration (data not shown). The DLMO time was calculated for each participant who exhibited a clear rhythm in melatonin. There was no significant (*p*>0.05; 1-way ANOVA) difference in DLMO between the groups (Figure 1b).

As a melatonin rhythm could not be detected in two of the seven T2DM participants, we analysed plasma cortisol concentrations in this subject group to determine whether the absence of melatonin rhythmicity was reflected in other SCN-driven endocrine rhythms (Figure S4). Six of the seven T2DM subjects, including both that lacked detectable melatonin rhythms, exhibited plasma cortisol rhythms, as determined by significant (*p*<0.05) correlation of cosinor curves with their plasma cortisol data.

**Figure 2:**
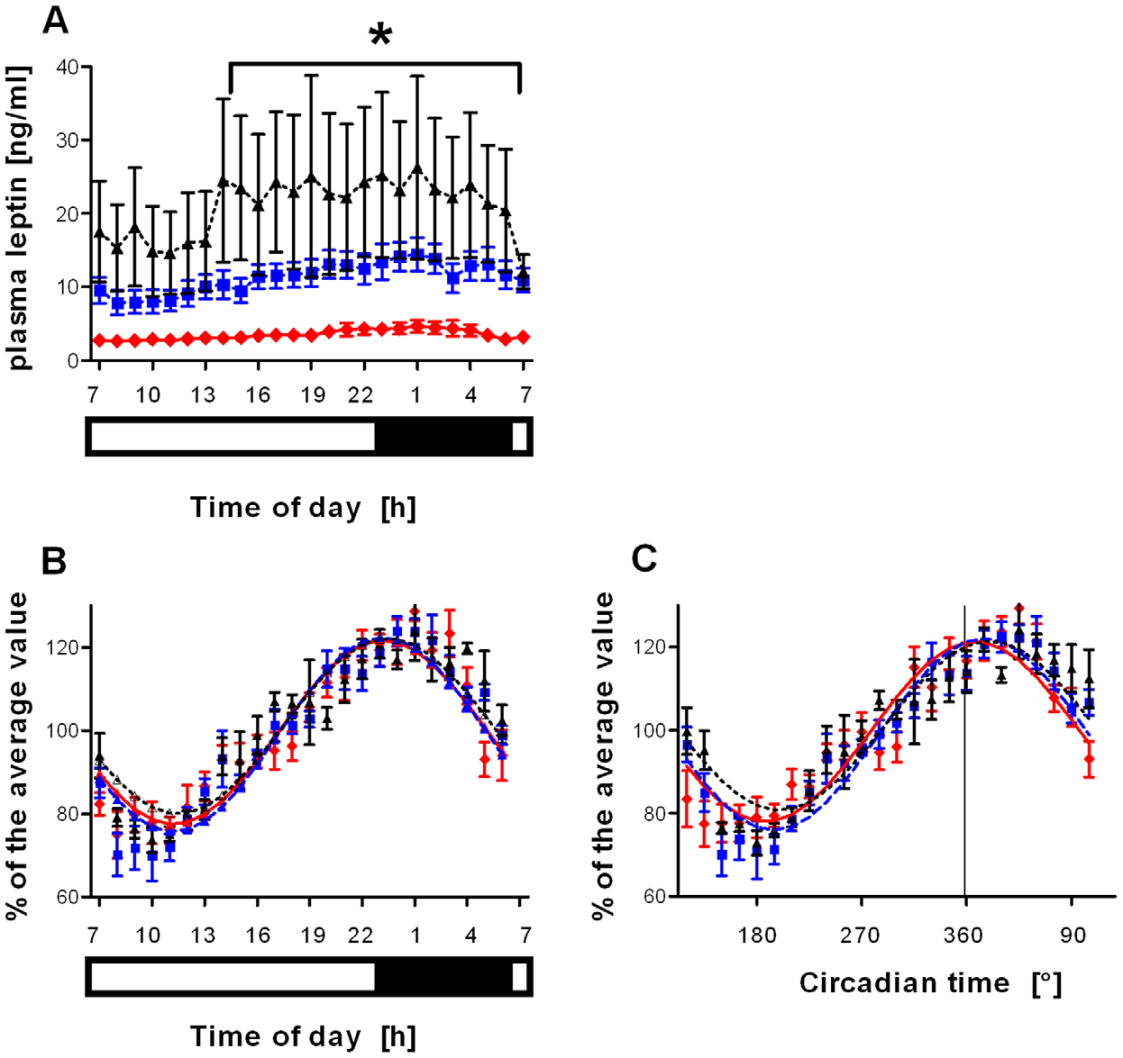
Diurnal rhythms of plasma leptin concentrations. (a) Analysis of absolute concentration revealed a significant effect of group (*p*<0.001; 2-way repeated measures ANOVA) but not of time or time x group interaction. *p<0.05 lean vs type 2 diabetic subjects. (b–c) Following normalisation of each individual’s raw data to their own mean concentration, the group values were calculated and fitted with a cosinor curve. Normalised data are expressed relative to (b) external time of day and (c) endogenous circadian time, estimated using DLMO where 360°  =  time of DLMO. The DLMO of two participants in the type 2 diabetic participant group could not be calculated due to the absence of a peak in the melatonin profile; their data were thus excluded. Statistical analysis showed a significant effect of time (*p*<0.001; 2-way repeated measures ANOVA) but not for group or interaction in both (b) and (c). (a–b) The light-dark conditions are indicated by the bars below the x-axes. In all panels, diamonds, solid red line  =  lean subjects (n = 8); square, dashed blue line  =  obese non-diabetic subjects (n = 10); triangle, dotted black line  =  type 2 diabetic subjects (n = 7).

There was a significant effect of group (*p*<0.001; 2-way repeated measures ANOVA) but neither time nor time x group interaction on raw plasma leptin concentrations (Figure 2a). Cosinor curves were fitted to all the individual leptin profiles and significant (*p*<0.05) rhythms were observed for all participants (Figure S5, S6, and S7). A similar temporal pattern of plasma leptin was observed in all subjects suggesting that the lack of effect of time in data shown in Figure 2a may be due to individual variation in basal leptin concentrations. Indeed, grouped plasma leptin data plotted as a percent of each individual’s mean leptin concentration revealed a clear 24-hour rhythm, with a nadir in the morning and peak concentration in the early night (Figure 2b–c). When plotted as a function of external clock time, there was a significant effect of time (*p*<0.001, 2-way repeated measures ANOVA) but neither group nor time x group interaction on leptin concentrations (Figure 2b). We therefore also plotted the leptin values following correction to each individual’s internal circadian phase, estimated by the DLMO of the participant (Figure 2c). Two way repeated measures ANOVA analysis again showed a significant effect for time (*p*<0.001) but neither for group nor time x group interaction. One-way ANOVA analysis revealed that there was no significant effect on either the timing or amplitude of the plasma leptin rhythms between groups (Table 2).

**Figure 3:**
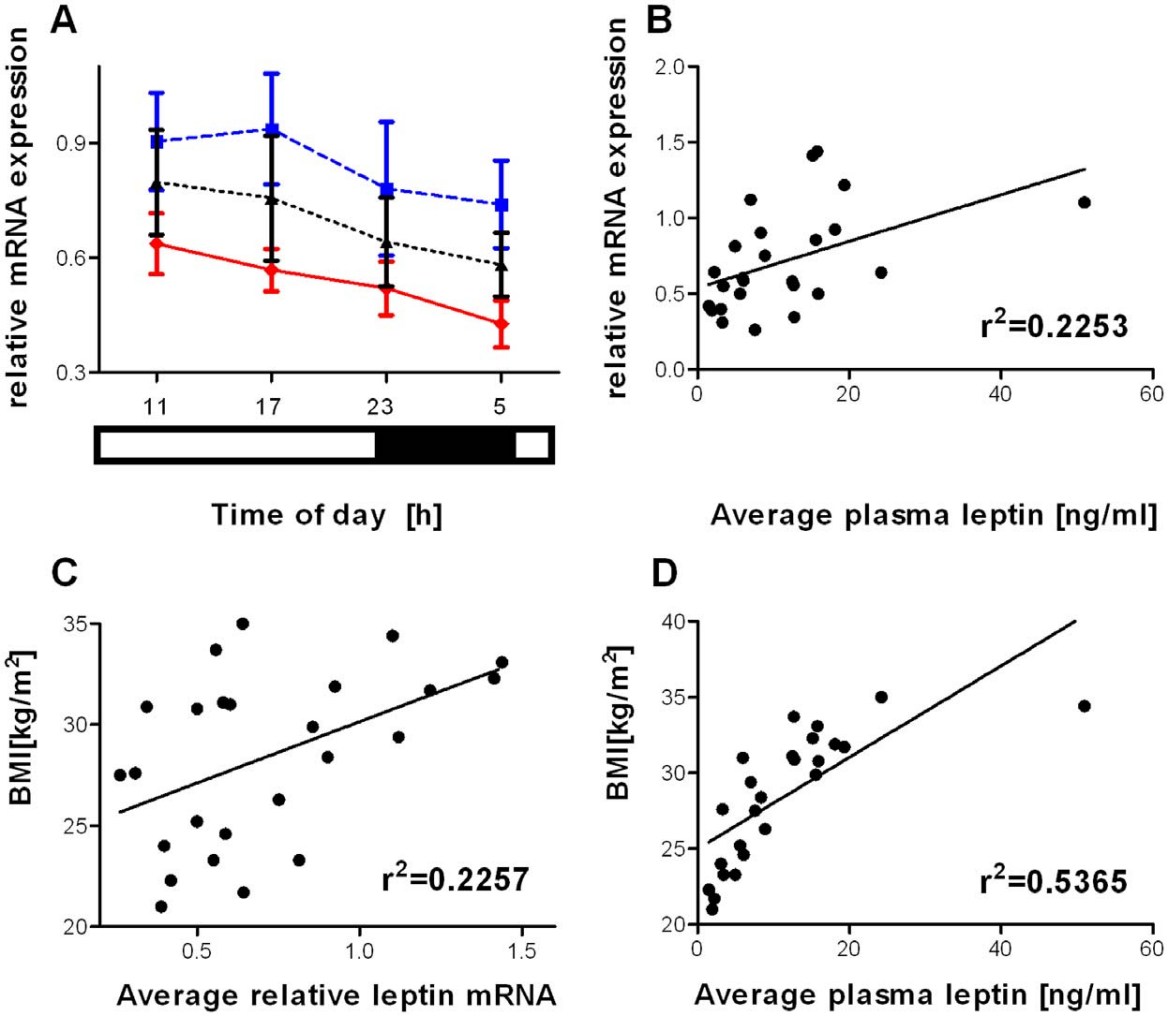
Expression of leptin mRNA in white adipose biopsies. (a) Data represent mean ± SEM of leptin mRNA in 6-hourly serial biopsies. There was a significant effect of group (*p*<0.05; 2-way repeated measures ANOVA), but not of time or time x group interaction. There were no significant (*p*>0.05; Bonferroni post-hoc test) pair wise differences in leptin mRNA expression between the subject groups. The light-dark conditions are indicated by the bars below the x-axis. Diamonds, solid red line  =  lean subjects (n = 8); squares, dashed blue line  =  obese non-diabetic subjects (n = 10); triangles, dotted black line  =  type 2 diabetic group (n = 7). The average leptin mRNA expression for each subject was significantly (*p*<0.05) correlated with both (b) average plasma leptin concentrations and (c) subjects’ BMI. (d) The average plasma leptin concentration for each subject was significantly (*p*<0.001) correlated with subjects’ BMI.

Expression of leptin mRNA in the WAT biopsies exhibited a significant (*p*<0.05; 2-way repeated measures ANOVA) overall effect of group, but no significant effect of time nor time x group interaction (Figure 3a). Post-hoc analysis failed to reveal a significant difference in leptin mRNA between the participant groups. The 24-hour mean expression of leptin mRNA in each subject significantly correlated with both 24-hour mean plasma leptin concentrations (Figure 3b; *p*<0.05; r^2^ = 0.2253) and BMI (Figure 3c; *p*<0.05; r^2^ = 0.2257). As expected, there was also a significant correlation between 24-hour mean plasma leptin concentration and BMI (Figure 3d; *p*<0.001; r^2^ = 0.5365).

**Table 2:** Acrophase (peak time) and amplitude of the leptin rhythms determined by cosinor analysis.

Group	N	Not DLMO correctedacrophase [h ± min]	DLMO corrected	Amplitude [% of mean]
Lean healthy	8	00∶04 15	00∶06 22	21.7 1.9
Obese non-diabetic	10	00∶16 16	00∶34 28	23.3 2.7
Type 2 diabetic	7	00∶31 26	00∶49 38	22.2 0.7

A cosine wave was fitted to each individual leptin profile. There was no significant (*p*>0.05; 1-way ANOVA) effect of group on either the acrophase (peak time) or amplitude of the rhythms. The acrophase of the leptin rhythm was also corrected to the dim light melatonin onset (DLMO).

## Discussion

This study revealed significantly higher nocturnal plasma melatonin concentrations in obese non-diabetic subjects than in weight-matched T2DM counterparts or lean, non-diabetic individuals. As expected, plasma leptin concentrations were elevated in obese non-diabetic and obese T2DM groups, compared to lean controls. Consistent with this finding, there was an overall significant effect of subject group on subcutaneous leptin mRNA measured in serial subcutaneous WAT biopsies. Although we were unable to detect a significant effect of time on either leptin mRNA expression or raw plasma leptin data, reproducible rhythms of plasma leptin were apparent when the data of each individual were normalised relative to his 24-hour mean. There was no significant effect of subject group on these daily rhythms of plasma leptin, either when expressed relative to external clock time or to internal circadian time as assessed by DLMO.

Melatonin rhythms provide an extremely robust endocrine marker of internal circadian time that is routinely used in human chronobiology. The absence of a change in DLMO between our subject groups implies that obesity and T2DM do not alter the phasing of the master circadian clock when studied in controlled laboratory conditions. The altered amplitude of the melatonin rhythm observed in our non-diabetic participants is consistent with previous work that reported a correlation between nocturnal melatonin concentrations and body weight in human subjects without diabetes [23]. The mechanism driving the increased melatonin rhythm amplitude in obese non-diabetic subjects is not known. However, increased obesity and leptin concentration are associated with increased sympathetic tone in some tissues [24], [25], indicating that altered sympathetic innervation of the pineal gland may underlie the increased melatonin concentration in our obese non-diabetic subjects.

By contrast, our T2DM subjects, who were matched with the obese non-diabetic participants for BMI and waist circumference, displayed significantly lower nocturnal melatonin concentration than the obese non-diabetic group. Indeed, two T2DM subjects failed to exhibit a detectable concentration of melatonin across the 24-hour cycle. These two subjects, however, showed normal cortisol rhythmicity and thus the absence of a melatonin rhythm is likely caused by an impaired clock output, rather than an impaired SCN clock. Although there was no significant difference in plasma melatonin between the lean and T2DM groups, this comparison is less meaningful than the comparison between weight-matched obese non-diabetic and T2DM groups, due to the effects of obesity per se described above. Reduced amplitude melatonin rhythms have been previously reported in T2DM patients with autonomic neuropathy [26], [27] and retinopathy [28]. A further study has reported a small decrease in serum melatonin across the 24-hour diurnal cycle in T2DM patients [29]; however, many of these individuals were extremely obese (mean BMI  = 44 for T2DM versus 34 for non-T2DM ) and there were no other participant details provided, making interpretation of the data extremely difficult. The findings from our study suggest that, even in well controlled T2DM patients with no symptoms of autonomic neuropathy, melatonin rhythms are blunted relative to BMI-matched individuals who exhibit normal insulin sensitivity.

Altered melatonin rhythm amplitude in our subjects may be functionally related to changes in both insulin secretion and sensitivity. Multiple studies have shown that melatonin can acutely inhibit glucose-mediated insulin secretion *in vitro* and *in vivo* [30]. In addition, 9 week nocturnal melatonin administration to rats via drinking water decreased plasma insulin concentration [31]. In rodent models, loss of melatonin signalling by pinealectomy [32] or genetic ablation of MT1 melatonin receptor expression [33] decreases insulin sensitivity. Although translation of data between nocturnal rodents and diurnal humans has limitations, altered melatonin signalling via polymorphism of the human melatonin MT2 receptor is associated with abnormal glucose homeostasis and T2DM [34]–[36]. This suggests that a functional link between melatonin signalling and diabetes is conserved between species. The intracellular mechanisms affected by altered melatonin amplitude in T2DM subjects are not yet known. Although the best characterised intracellular signalling pathway regulated by melatonin is cAMP synthesis, many other signalling mechanisms have been identified in a variety of model systems [37]. In a circadian context, it is of interest that melatonin regulates the expression of circadian clock genes in multiple tissues [38]–[41]. The relationship between plasma melatonin concentration and clock gene expression, however, is not clearly defined. Moreover, there are multiple SCN-derived pathways that regulate peripheral clocks [3]. It is therefore unlikely that the changes in melatonin rhythm amplitude observed here would result in physiologically relevant changes in most peripheral rhythms.

Soon after the seminal discovery of leptin as an adipose hormone [42], it was reported that human plasma leptin concentrations exhibit a 24-hour variation. Although these rhythms are influenced by feeding and time awake, an endogenous circadian component has been observed [14], which may in part be a result of circadian secretion from adipocyte cells [15]. Inconsistencies in 24-hour plasma leptin profiles and their modulation by obesity and T2DM have been reported in the literature [16]–[18]. These differences may be attributable to a lack of circadian control prior to the laboratory study and also differences in the BMI of participants within the obese subject groups. Our study thus employed carefully controlled conditions to investigate the effect of metabolic status while minimising the confounding effects of lifestyle factors and different circadian phases. The subjects recruited into our obese non-diabetic and T2DM groups were not morbidly obese. Moreover, although HOMA-IR and HbA1c values for the T2DM subjects were significantly higher than that of the other groups, they were sufficiently low to indicate that our subjects’ diabetes was well controlled. Data may therefore be different in individuals with greater levels of obesity and poorly controlled diabetes; however, this was not the focus of the current study and our data clearly suggest that obesity and T2DM *per se* do not alter diurnal rhythms of plasma leptin concentration. The possibility that lifestyle changes or other factors tightly controlled in our study cause disruption of diurnal and/or circadian rhythmicity remains to be determined.

Analysis of leptin mRNA expression in serial subcutaneous WAT biopsies did not reveal significant temporal variation. In mice, subcutaneous WAT also exhibits little temporal variation of leptin mRNA, despite high amplitude leptin mRNA rhythms in epididymal WAT [43]. It is therefore possible that temporal changes in leptin mRNA expression exist in other human WAT depots. The 24-hour mean expression of leptin mRNA in each of our subjects significantly correlated with both 24-hour mean plasma leptin and BMI. However, there was a much tighter correlation between 24-hour mean plasma leptin and BMI, suggesting that plasma leptin is a better indicator of adiposity.

In summary, our data reveal a correlation between nocturnal melatonin concentration and T2DM, which may support the possible existence of a functional link between altered melatonin production, obesity and insulin sensitivity. Our data do not support the hypothesis that obesity or T2DM influences 24-hour leptin rhythms in controlled laboratory conditions. Future work will study the mechanistic relationship between melatonin and glucose homeostasis.

## Supporting Information

Figure S1 **Individual plasma melatonin profiles and age of all the lean subjects.** The light-dark conditions are indicated by the bars below the x-axes.
(TIF)

Figure S2 **Individual plasma melatonin profiles and age of all the obese non-diabetic subjects.** The light-dark conditions are indicated by the bars below the x-axes.
(TIF)

Figure S3 **Individual plasma melatonin profiles, age and diabetes treatment regimes of all the type 2 diabetic subjects.** The light-dark conditions are indicated by the bars below the x-axes.
(TIF)

Figure S4 **Plasma cortisol profiles of the type 2 diabetic subjects.** Six out of seven subjects exhibited a plasma cortisol rhythm as determined by significant (*p*<0.05) cosine fit to the data. The light-dark conditions are indicated by the bars below the x-axes. The top left and right panels show cortisol rhythms in subjects that did not exhibit plasma melatonin rhythms.
(TIF)

Figure S5 **Individual plasma leptin profiles of all the lean subjects.** The light-dark conditions are indicated by the bars below the x-axes. Cosinor curve fits are shown for each profile.
(TIF)

Figure S6 **Individual plasma leptin profiles of all the obese non-diabetic subjects.** The light-dark conditions are indicated by the bars below the x-axes. Cosinor curve fits are shown for each profile.
(TIF)

Figure S7 **Individual plasma leptin profiles of all the type 2 diabetic subjects.** The light-dark conditions are indicated by the bars below the x-axes. Cosinor curve fits are shown for each profile.
(TIF)
