# Supplementary figures and images for: Daily Rhythms of Plasma Melatonin, but Not Plasma Leptin or Leptin mRNA, Vary between Lean, Obese and Type 2 Diabetic Men
Source: PLoS One. 2012 May 18;7(5):e37123. doi: 10.1371/journal.pone.0037123 (PMC3356389; doi:10.1371/journal.pone.0037123)

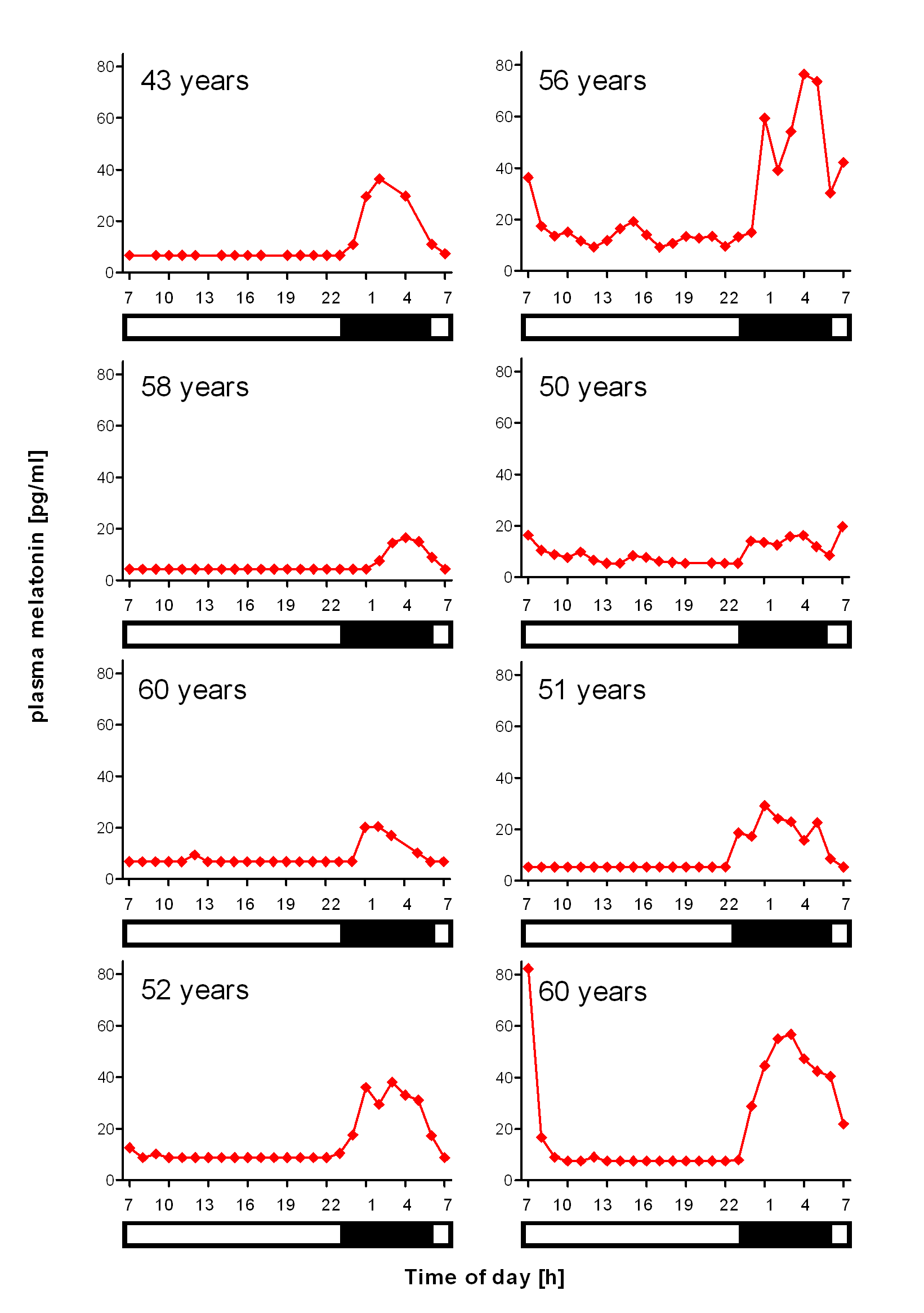

Supplement: Figure S1 — Individual plasma melatonin profiles and age of all the lean subjects. The light-dark conditions are indicated by the bars below the x-axes. (TIF) [file pone.0037123.s001.tif]

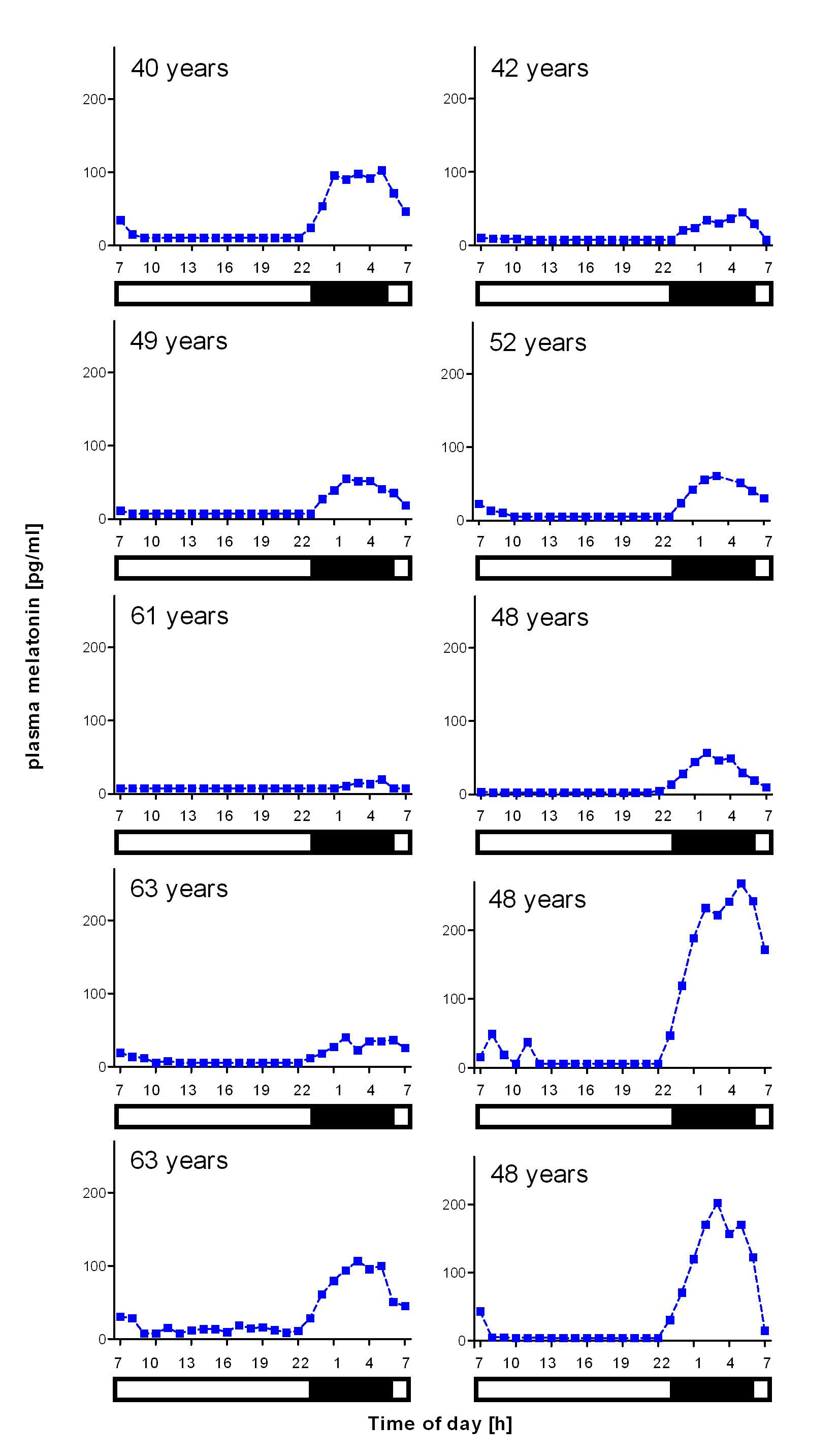

Supplement: Figure S2 — Individual plasma melatonin profiles and age of all the obese non-diabetic subjects. The light-dark conditions are indicated by the bars below the x-axes. (TIF) [file pone.0037123.s002.tif]

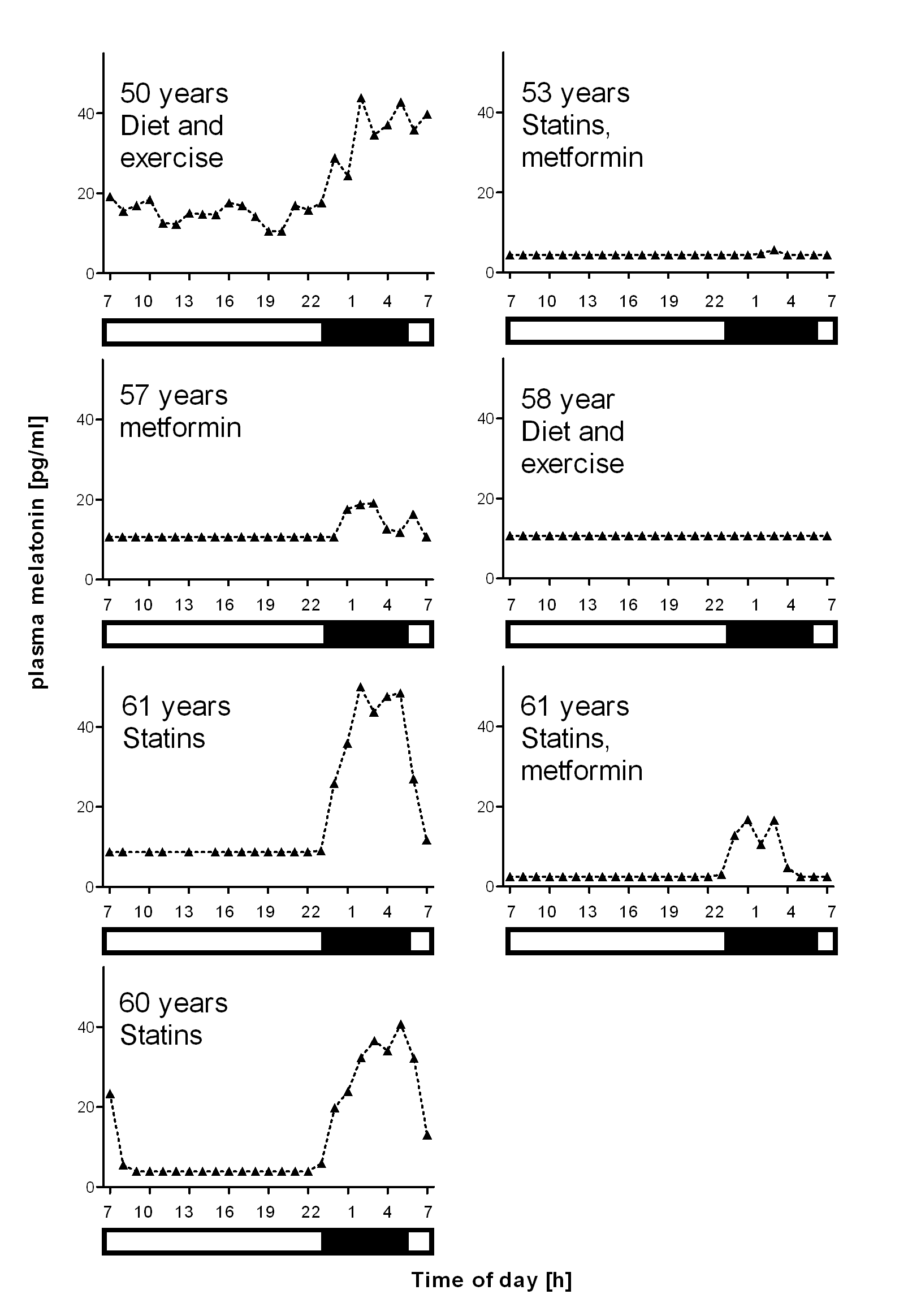

Supplement: Figure S3 — Individual plasma melatonin profiles, age and diabetes treatment regimes of all the type 2 diabetic subjects. The light-dark conditions are indicated by the bars below the x-axes. (TIF) [file pone.0037123.s003.tif]

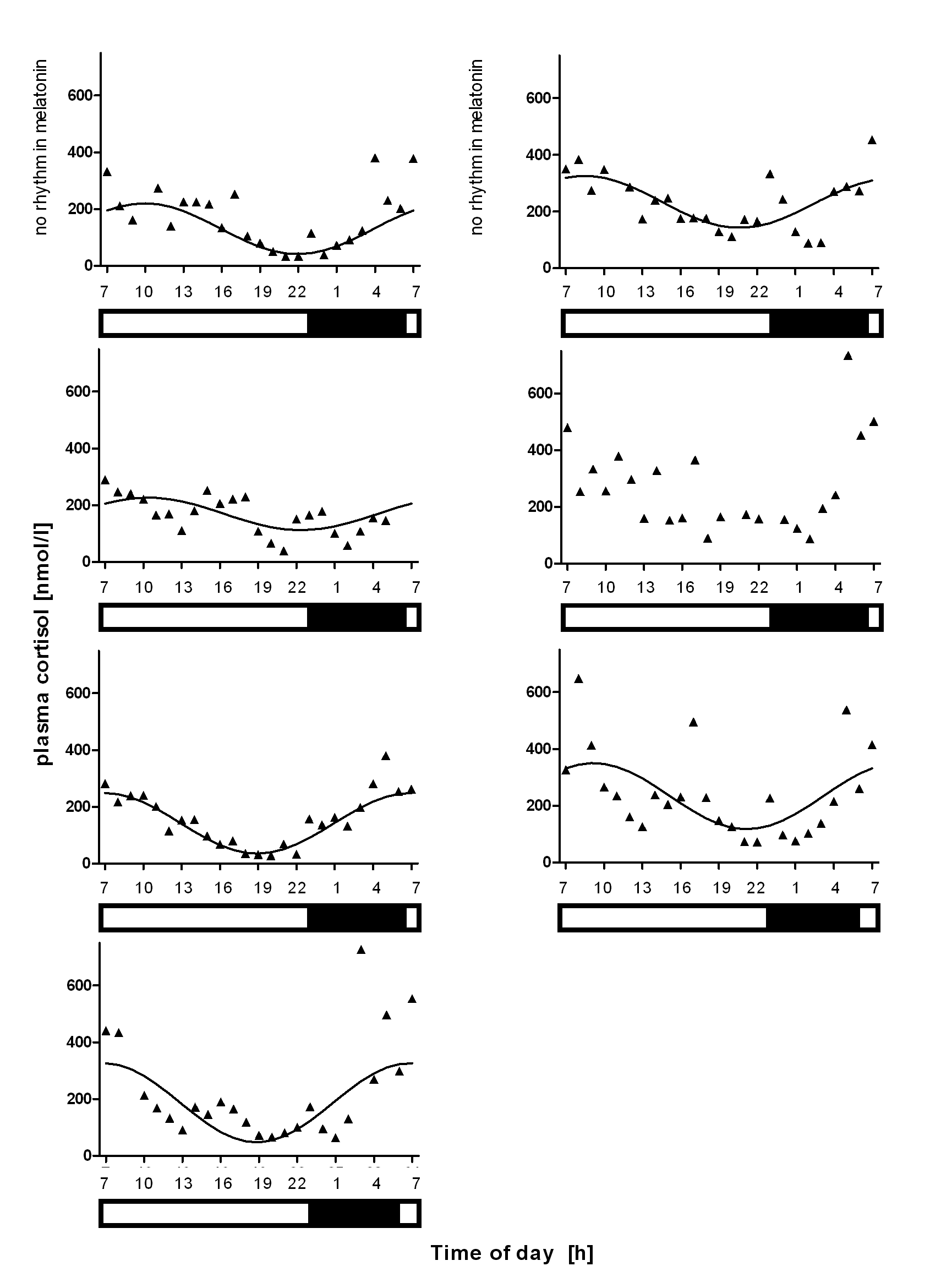

Supplement: Figure S4 — Plasma cortisol profiles of the type 2 diabetic subjects. Six out of seven subjects exhibited a plasma cortisol rhythm as determined by significant (p<0.05) cosine fit to the data. The light-dark conditions are indicated by the bars below the x-axes. The top left and right panels show cortisol rhythms in subjects that did not exhibit plasma melatonin rhythms. (TIF) [file pone.0037123.s004.tif]

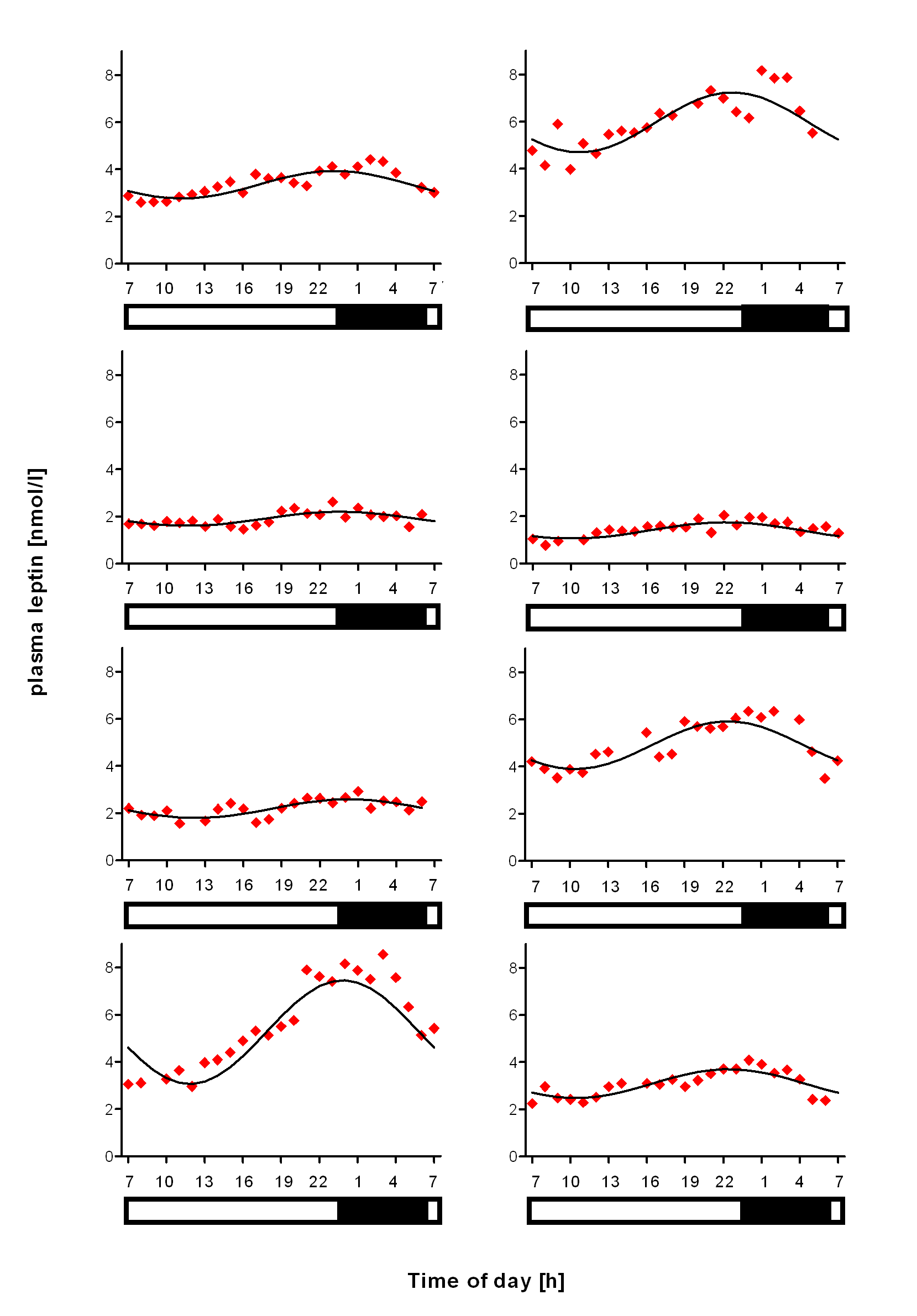

Supplement: Figure S5 — Individual plasma leptin profiles of all the lean subjects. The light-dark conditions are indicated by the bars below the x-axes. Cosinor curve fits are shown for each profile. (TIF) [file pone.0037123.s005.tif]

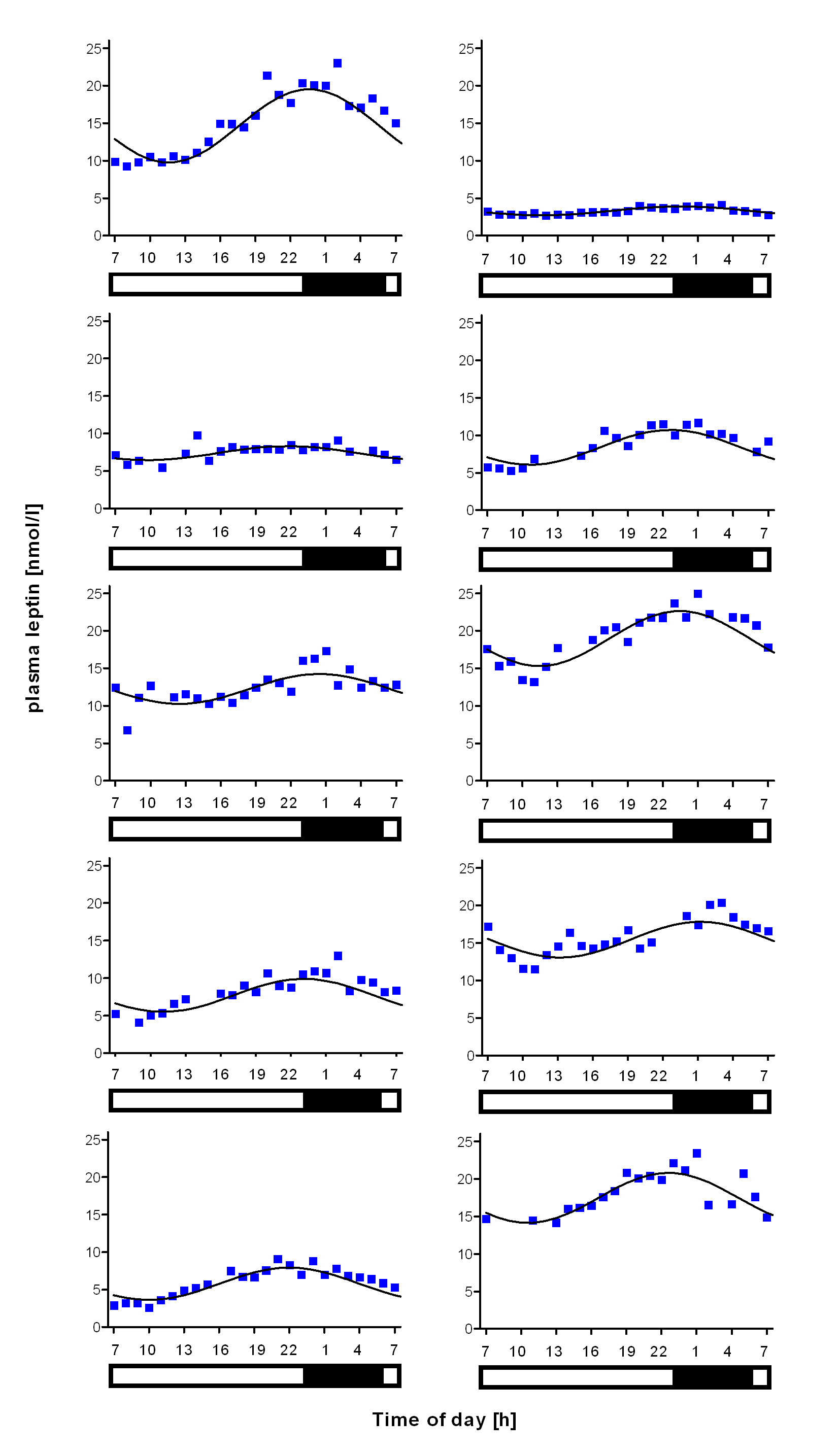

Supplement: Figure S6 — Individual plasma leptin profiles of all the obese non-diabetic subjects. The light-dark conditions are indicated by the bars below the x-axes. Cosinor curve fits are shown for each profile. (TIF) [file pone.0037123.s006.tif]

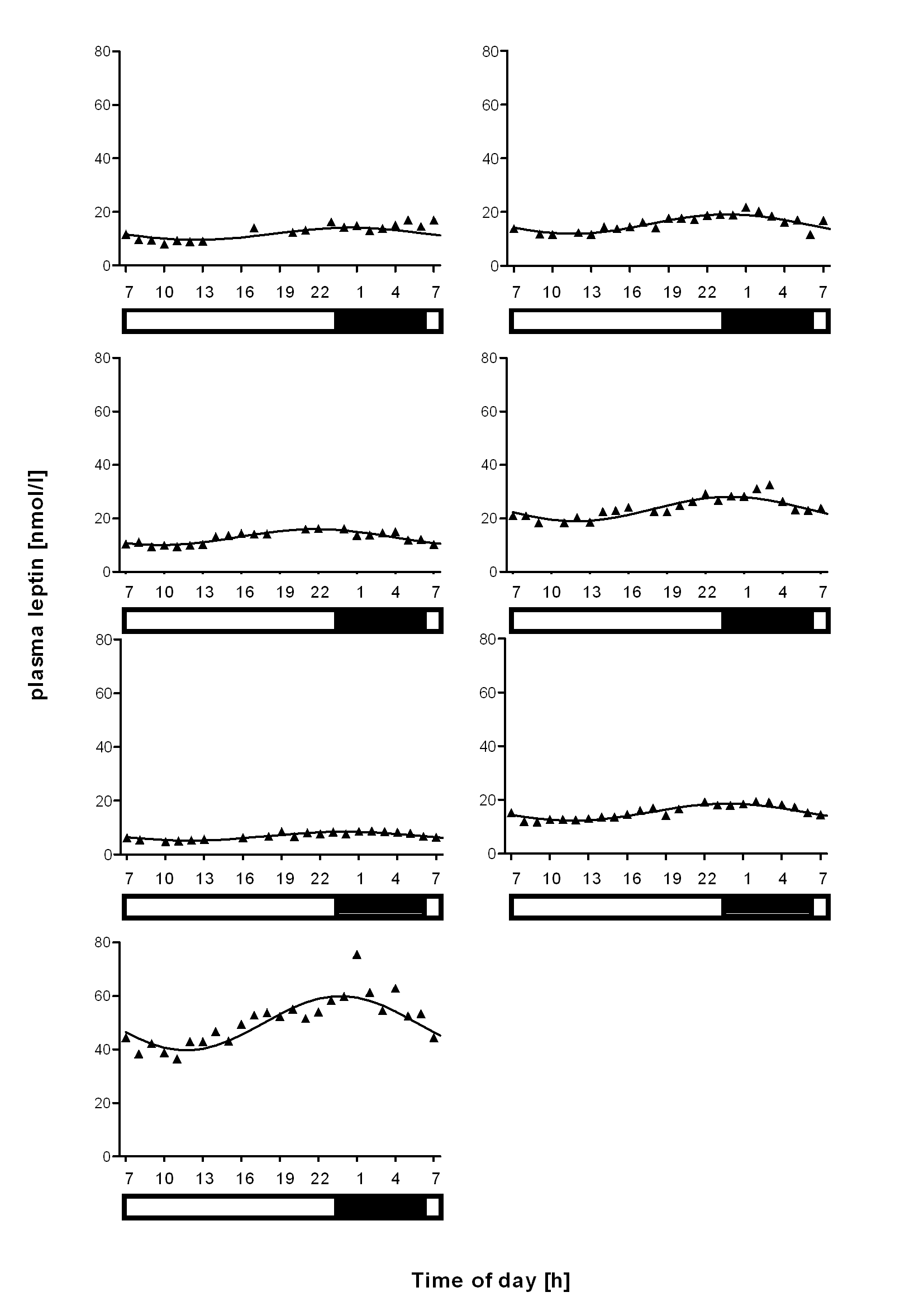

Supplement: Figure S7 — Individual plasma leptin profiles of all the type 2 diabetic subjects. The light-dark conditions are indicated by the bars below the x-axes. Cosinor curve fits are shown for each profile. (TIF) [file pone.0037123.s007.tif]
